# The impact of dementia on the use of general practitioners among the elderly in Norway

**DOI:** 10.3109/02813432.2015.1067516

**Published:** 2015-07

**Authors:** A.E. Ydstebø, S. Bergh, G. Selbæk, J. Šaltytė Benth, H. Lurås, C. Vossius

**Affiliations:** 1 Centre for Age-related Medicine, Stavanger University Hospital, Norway; 2 Centre for Development of Institutional and Home Care Services Rogaland, Norway; 3 Centre for Old Age Psychiatry Research, Innlandet Hospital Trust, Norway; 4 Helse Sør-Øst Health Services Research Centre, Akershus University Hospital, Norway; 5 Institute of Clinical Medicine, University of Oslo; 6 Norwegian National Advisory Unit on Ageing and Health, Vestfold Hospital Trust, Tønsberg, Norway

**Keywords:** Dementia, emergency service, general practice, general practitioner, municipal care, Norway

## Abstract

*Objective.* To assess the use of general practitioners (GPs), in elderly home-dwelling persons in Norway and explore the impact of cognitive decline, age, and living situation. *Design.* Prospective longitudinal study. *Setting.* Data were collected from municipalities in four counties in Norway in the period from January 2009 to August 2012. *Subjects.* Home-dwelling persons 70 years of age or older, receiving in-home care. *Main outcome measures.* Use of GPs over a period of 18 months related to cognitive state, functional status, neuropsychiatric symptoms, and demographics. *Results.* A total of 599 persons were included. The mean annual number of consultations per participant was 5.6 (SD = 5.4). People with moderate to severe dementia had fewer consultations per year compared with those with mild or no dementia (3.7 versus 5.8 per year, p = 0.004). In the multivariate model higher age predicted fewer consultations while affective neuropsychiatric symptoms were associated with an increase in frequency of consultations. The most frequent reason to consult a GP was cardiovascular diseases (36.8% of all consultations), followed by musculoskeletal complaints (12.1%) and psychiatric diagnoses (8.7%). *Conclusion.* Our study shows that the home-dwelling elderly with moderate to severe dementia in Norway consult their GP less often than persons with mild or no dementia. This could indicate a need for better interaction between the municipal care and social services and the general practitioners.

- People with moderate to severe dementia had fewer consultations with their GP per year compared with those with mild or no dementia.
- Increased age predicted fewer consultations with the GP while affective neuropsychiatric symptoms were associated with an increase in frequency of consultations.
- The findings could indicate a need for better interaction between the municipal care and social services and the general practitioners.

## Introduction

It has been estimated that around 70 000 Norwegians are suffering from dementia [1] and that about half of them are living at home [2]. A British study showed that 89% of those with dementia had at least one comorbid condition and that 57% were multimorbid [3]. A reasonable assumption would be that this is a group of high-frequency users of primary and specialist health care services, although the literature suggests otherwise, showing that dementia is associated with an increase in the use of municipal care and social services but no increase in the use of general practitioners (GPs) [4,5].

Norway has a list-patient system in general practice where every inhabitant is registered with a GP, and the GPs serve as gatekeepers for the specialist health care services. During weekends and outside business hours patients are entitled to contact municipal emergency units when they need a medical consultation. GPs are crucial for the coordination of municipal care services, and social services rendered by the municipality. In elderly home-dwelling persons with dementia, the GP is supposed to have a key role, working together with the municipal health care services in assessment, diagnosis, treatment, and follow-up.

There are several studies exploring differences in treatment by GPs of patients with and without dementia. Two studies that explored differences between persons with and without dementia, and the use and prescription of cardiovascular medication, found that persons with dementia were less likely to use lipid-lowering drugs [6], and were prescribed fewer cardiovascular medications than the non-demented group [7]. A third study found little evidence supporting differences in treatment of diabetes, hypertension, and hyperlipidaemia between the two groups [8]. In Sweden a dementia-management programme involving GPs and community nurses in early diagnosing of dementia and drug evaluation were successful in increasing the number of persons diagnosed with dementia and appeared to improve the management of psychotropic drugs [9,10]. However, few studies have examined the behaviour of patients and how they seek contact with their GP. In order to enable the GP to follow the patient's course of dementia and to coordinate services from both specialist health care and municipal health and social care, an increasing number of contacts with the GP is expected.

The aim of this study was to assess the use of GPs in respect of elderly home-dwelling persons. We aimed at exploring the impact of cognitive decline, age, and living situation on the frequency of contacts with the GP, and whether fewer visits to the GP were related to an increased number of visits to the municipal emergency service.

## Material and methods

### Study population

Patients were drawn from a prospective longitudinal study including 1001 home-dwelling persons aged 70 years or older receiving municipal services such as home care, cleaning help, meals-on-wheels, day care centre, municipal housing, or a safety alarm at baseline, and where both the client and a proxy were willing to participate in the study [11]. Assessments were carried out by trained health care-workers and the patients were examined at baseline and approximately 18 months later. Baseline inclusion was from January 2009 to August 2010, and the last follow-up examinations were performed in August 2012. A detailed explanation on the data collection can be found in Wergeland et al. [11]. Of the 1001 participants included at baseline, 599 attended the follow-up examination. In the present study, only those who attended both examinations were included. The 402 patients who did not attend the follow-up examination were excluded. In detail, these dropouts were due to the following: as a consequence of a revised study protocol for the follow-up examination new written consent of all participants was necessary and 146 (14.6%) persons did not consent to follow-up. In total 180 persons (18.0%) died and two (0.2%) moved out of the area. Seventy-four (7.4%) had other reasons; for most of them an examination was not possible within the required timeframe. As compared with the included patients those who dropped out of the study were slightly older (mean age of 83.9 [SD = 5.6] versus 83.0 [SD = 5.4] years; p = 0.013), fewer were females (64.4% versus 70.8%; p = 0.038), and they had lower functioning in instrumental activities of daily living (IADL) (mean score of 0.66 [SD = 0.30] versus 0.71 [SD = 0.30], p = 0.017) (Table I).

**Table I.** Study population: Demographics, physical health, and cognitive state at baseline.

	Patients included in the study (n = 599)	Patients not included in the study (n = 402)	p-value
Age			
n	599	402	0.010^1^
Mean (SD)	83.0 (5.6)	83.9 (5.8)	
Gender			
n	599	402	0.038^2^
Male, n (%)	175 (29.2)	143 (35.6)	
Female, n (%)	424 (70.8)	259 (64.4)	
Living situation			
n	588	396	0.188^2^
Alone, n (%)	407 (69.2)	258 (65.2)	
With others, n (%)	181 (30.8)	138 (34.8)	
MMSE			
n	590	392	0.173^1^
Mean (SD)	24.6 (4.7)	24.2 (5.1)	
IADL			
n	579	387	0.017^1^
Mean (SD)	0.71 (0.3)	0.66 (0.30)	
CDR			
n	590	398	0.678^1^
Mean (SD)	0.56 (0.62)	0.58 (0.67)	
GMHR			
n	575	373	0.001^3^
Poor, n (%)	46 (8.0)	54 (14.5)	
Fair, n (%)	194 (33.7)	135 (36.2)	
Good, n (%)	230 (40.0)	143 (38.3)	
Excellent, n (%)	105 (18.3)	41 (11.0)	
Diagnosis of dementia			
n	598	402	
No dementia	185 (30.9)	123 (30.6)	
MCI	172 (28.8)	105 (26.1)	0.506^3^
Dementia	241 (40.3)	174 (43.3)	
Agitation			
n	574	392	0.390^1^
Mean (SD)	1.5 (3.8)	1.7 (5.1)	
Psychosis			
n	581	394	0.243^1^
Mean (SD)	0.6 (2.2)	0.4 (1.6)	
Affective			
symptoms			
n	577	395	0.980^1^
Mean (SD)	2.9 (5.4)	2.9 (5.1)	

Notes: MCI = mild cognitive impairment; SD = standard deviation; MMSE = Mini Mental State Examination; IADL = instrumental activities of daily living; CDR = Clinical Dementia Rating; GMHR = General Medical Health Rating scale. ^1^Independent-samples t-test. ^2^Fisher's exact test. ^3^Chi-square-test.

The demographic and clinical data from the 599 elderly included in the study were merged with data on the use of primary health care services (both the use of GPs and the use of emergency services) from the Norwegian Health Economics Administration, the public agency responsible for the reimbursement of primary care services in Norway.

In addition to demographic data, data from the following four clinical assessments were collected. Evaluation of physical health was performed by the General Medical Health Rating (GMHR) scale [12], which rates health into the four categories poor = 0, fair = 1, good = 2, and excellent = 3 according to the rater's overall impression. Evaluation of functional status was carried out by the Lawton IADL Scale [13], which comprises the eight items “ability to use telephone”, “shopping”, “food preparation”, “housekeeping”, “laundry”, “mode of transportation”, “responsibility for own medications”, and “ability to handle finances”. Each item can be scored “0” (dependent) or “1” (independent). For women, all eight items were included in the sum score, while we excluded the items “food preparation”, “housekeeping”, and “laundry” for men, as these items were not applicable for many male participants in this study [13,14]. We calculated a sum score and divided it by the number of items evaluated, thus obtaining a score ranging from 0 = completely dependent to 1 = completely independent in terms of IADL. The cognitive state was assessed by the Mini Mental State Examination (MMSE) ranging from 0 to 30, where a score of 30 indicates unimpaired cognitive functioning [15]. A dementia staging was performed using the six-item Clinical Dementia Rating (CDR) Scale [16] with the stages no dementia = 0, possible dementia = 0.5, mild dementia = 1, moderate dementia = 2, and severe dementia = 3, based on an algorithm giving precedence to the item memory. In addition, the CDR sum of boxes was calculated as described in previous publications [17]. In the present material, CDR and CDR sum of boxes correlated highly (Spearman correlation coefficient of 0.93). Further, an evaluation of whether the participant was without cognitive impairment, had a minimal cognitive impairment according to the Winblad criteria [18], or had dementia according to the ICD-10 criteria [19] at baseline was made independently by two experts (GS and SB) based on all available clinical information. Neuropsychiatric symptoms were evaluated by the Neuropsychiatric Inventory (NPI) [20], 10-item version. The frequency (0–4) and intensity (0–3) of each item are multiplied to produce an item score of 0–12. We identified three sub-syndromes of the NPI based on a principal component analysis with direct oblimin rotation. The components were extracted based on the Kaiser criterion (factors with eigenvalues under 1 are dropped) and inspection of the screenplot. We termed the sub-syndromes “Agitation”, “Psychosis”, and “Affective symptoms”. “Agitation” was composed of the items agitation/aggression, euphoria, disinhibition, aberrant motor behaviour, and irritability; “psychosis” was composed of the items delusions and hallucinations; and “affective symptoms” was composed of the items depression, anxiety, and apathy. The item agitation/aggression loaded also on the “Psychosis” sub- syndrome, but in line with previous research and clinical experience [21] we chose to include it in the “Agitation” sub-syndrome.

### Use of general practitioners and municipal emergency service

For every participant, data on the use of GP and municipal emergency services between 1 January 2009 and 31 December 2012 were provided by the Norwegian Health Economics Administration. The following information was provided: date of contact, whether the GP or the municipal emergency service was contacted, and the diagnoses causing the contact registered according to the International Classification of Primary Care, version 1 (ICPC-1). Only consultations during the period between baseline and follow-up examination were registered. To adjust for varying intervals between these two examinations, we calculated “consultations per year” by dividing the total number of consultations by the length of the observation period in years for each individual. This includes home visits. For participants who were admitted to a nursing home during the observation period, we considered the length of the observation period to be from baseline until nursing home admission. This is due to the fact that when patients move into a nursing home they are no longer followed up by their GP, but by nursing home doctors. Contacts by phone were evaluated as well. However, our findings added no information to the data presented in the article, and were therefore not included in the presentation of results.

### Statistics

The program SPSS^™^ 22.0 (SPSS Inc, Chicago, USA) was used for statistical analysis. Demographic and clinical characteristics at baseline were presented as means and standard deviations (SD) or frequencies and percentages, as appropriate. Comparison of those included versus not included in the study as well as those who did not visit their regular GP versus those who had at least one consultation per year was performed by an independent-samples t-test for continuous variables and chi-square or Fisher's exact test for categorical variables. The distribution of the number of consultations with the GP was skewed. As ln-transformation was not appropriate due to many zeroes, the variable was categorized to 0 (0–2, 2–4, 4–7), and > 7 consultations. To assess the relationship between the categorized number of consultations with the GP and demographic and clinical characteristics of patients, the bivariate ordinal regression model was estimated first. The following patient characteristics were included in the analysis: age, gender, living situation, CDR sum of boxes, IADL, and the neuropsychiatric sub-syndromes “agitation”, “psychosis”, and “affective symptoms”. Next, a multivariate ordinal regression model with all considered patient characteristics was estimated. Finally, the multivariate model was adjusted for confounder, GMHR. A test of parallel lines was applied to assess the assumption for ordinal regression.

Two-sided p-values lower than 0.05 were considered statistically significant.

### Ethics

The regional ethics committee (registration number 2010/119) approved the study. All participants gave informed written consent.

## Results

### Study population

A total of 599 participants with a mean age of 83.0 (SD = 5.6) years were included; 175 (29.2%) were males. Table I contains the demographic characteristics, physical health, and cognitive state of patients included.

### Consultations with the GP

**Figure 1.**
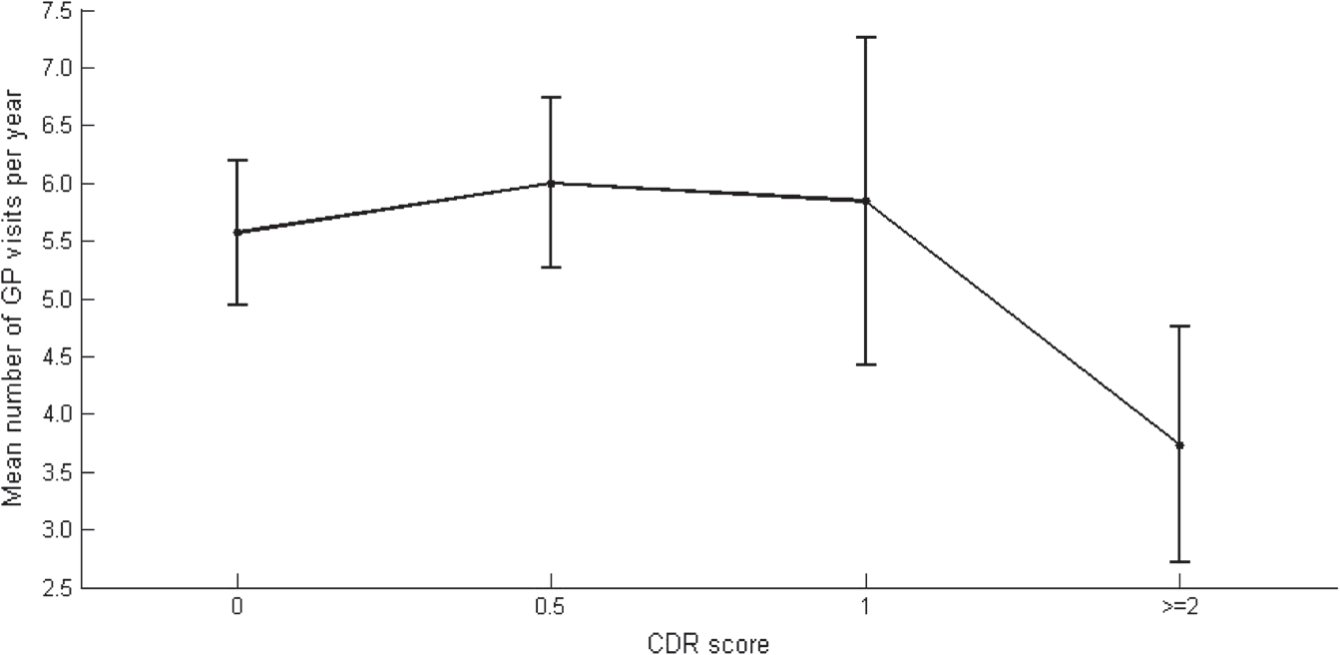
GP visits per year related to CDR score. Notes: CDR 0: n = 214; CDR 0.5: n = 212; CDR 1: n = 104; CDR 2: n = 57; CDR 3: n = 3; missing = 9. CDR 2 and CDR 3 are combined into one category due to low number of patients with CDR3. CDR = Clinical Dementia Rating, GP = general practitioner.

The mean number of consultations per year and participant were 5.6 (SD = 5.4). People with moderate to severe dementia had fewer consultations with their GP per year compared with those with mild or no dementia (3.7 versus 5.8 times per year, p = 0.004). Figure 1 illustrates the association between the CDR score and number of GP visits per year. Seventy-nine (13.2%) participants did not visit their GP at all during the observation period, making the distribution highly skewed. Number of consultations per year was therefore categorized into five different groups for further analysis (Table II).

**Table II.** Visits to the regular GP – categorization and number of persons per category.

Category	Number of visits to the GP per year	Number of persons (%)
1	0	79 (13.2)
2	(0–2]	72 (12.0)
3	(2–4]	120 (20.0)
4	(4–7]	158 (26.4)
5	> 7	170 (28.4)

Note: GP = general practitioner.

A bivariate ordinal regression analysis between the number of consultations per year and patient characteristics (Table III) showed that higher age, a decline in cognitive function (higher CDR sum of boxes score), and lower IADL functioning (lower score on Lawton & Brody's IADL scale) were associated with fewer consultations per year. Gender, living situation, “agitation”, “psychosis”, and “affective sub-syndrome” were not associated with the number of consultations per year. In a multivariate ordinal regression model, the number of consultations per year was statistically significantly associated with age and “affective sub-syndrome”, also after adjustment for GMHR. For a one-year increase in age, one can expect a 5% (p < 0.001) decline in odds of being in a higher category of number of consultations per year (OR = 0.95; CI 0.92, 0.97). However, for a one-unit increase in “affective sub-syndrome” score one can expect about 4% (p = 0.022) increase in odds of being in a higher category of number of consultations per year (OR = 1.04; CI: 1.00, 1.08).

**Table III.** Bivariate and multivariate ordinal regression analysis for frequency of GP consultations: odds ratios (ORs) with 95% confidence intervals (CI).

	Bivariate ordinal regression	Multivariate ordinal regression, unadjusted for confounder	Multivariate ordinal regression, adjusted for confounder
Variable	OR (95% CI)	p-value	OR (95% CI)	p-value	OR (95% CI)	p-value
Age	0.95 (0.93; 0.98)	**< 0.001**	0.95 (0.92; 0.98)	**< 0.001**	0.95 (0.92; 0.97)	**< 0.001**
Gender (ref. = men)	0.87 (0.64; 1.20)	0.398	0.89 (0.62; 1.28)	0.531	0.93 (0.64; 1.34)	0.697
Living alone (ref. = yes)	1.23 (0.90; 1.69)	0.189	1.23 (0.86; 1.75)	0.255	1.22 (0.85; 1.76)	0.272
CDR sum of boxes IADL mean	0.93 (0.90; 0.97) 2.42 (1.41; 4.15)	**< 0.001** **0.001**	0.97 (0.91; 1.04) 1.81 (0.72; 4.57)	0.420 0.208	0.97 (0.91; 1.04) 1.70 (0.65; 4.45)	0.454 0.283
Agitation	0.96 (0.93; 1.00)	0.055	0.97 (0.93; 1.01)	0.118	0.97 (0.93; 1.01)	0.124
Psychosis	0.96 (0.90; 1.02)	0.214	0.94 (0.86; 1.03)	0.168	0.93 (0.84; 1.02)	0.112
Affective symptoms	1.02 (0.99; 1.04)	0.261	1.04 (1.01; 1.08)	**0.012**	1.04 (1.00; 1.08)	**0.022**
GMHR:						
Poor	0.92 (0.50; 1.71)	0.797			0.95 (0.47; 1.93)	0.897
Fair	0.80 (0.52; 1.22)	0.302			0.95 (0.60; 1.51)	0.828
Good	0.93 (0.61; 1.40)	0.719			1.03 (0.67; 1.59)	0.884
Excellent = ref.	1	–			1	–

Notes: GP = general practitioner; OR = odds ratio; CI = confidence interval; CDR = Clinical Dementia Rating; IADL = instrumental activity of daily living; GMHR = General Medical Health Rating scale.

Compared with those who visited their GP during the observation period, patients who did not visit their GP had a more severe dementia measured with CDR sum of boxes 4.4 (SD = 4.6) versus 3.0 (SD = 3.6), (p = 0.013) and MMSE 23.5 (SD = 5.2) versus 24.8 (SD = 4.6), (p = 0.025). There were no differences regarding age, IADL functioning, neuropsychiatric sub-syndromes, gender, or living situation.

The most frequent reason to consult a GP was cardiovascular diseases, accounting for 36.8% of all consultations, followed by musculoskeletal complaints with 12.1% and psychiatric diagnoses with 8.7% of all consultations. The 10 most frequent diagnoses are shown in Table IV, the first four being atrial fibrillation (12.3%), hypertension (6.1%), dementia (5.2%), and diabetes (5.2%).

**Table IV.** The ten most frequent diagnoses for GP consultations.

Diagnosis	%
Atrial fibrillation	12.3
Hypertension	6.1
Dementia	5.2
Diabetes	5.2
Hip + knee arthrosis	4.1
Wounds	3.9
Heart failure	3.4
Stroke	3.3
Pulmonary infection	2.5
Urinary tract infection	2.2
Other	51.8

Note: GP = general practitioner.

### Consultations at the municipal emergency service

The mean number of consultations at the municipal emergency service was 0.6 (SD = 1.0) per patient per year. The three most frequent diagnoses causing the consultation with emergency service were gastrointestinal symptoms (11.3%), urinary tract infections (7.9%), and respiratory tract infections (7.2%). There were no correlation between the frequency of consultations with the GP and the frequency of consultations at the emergency service (Spearman correlation coefficient of 0.5).

## Discussion

The study evaluated the use of GP and municipal emergency services by an elderly home-dwelling population receiving municipal health and social care services. We found that people with moderate to severe dementia had fewer consultations with their GP per year compared with those with mild or no dementia. Further, we found that higher age resulted in fewer visits to the GP, while a higher burden of affective symptoms was associated with more frequent visits. It seems that the municipal emergency centre did not serve as a substitute for the use of GPs.

The strength of this study is the large cohort with participants including both rural and urban areas. The participants were assessed with a standardized protocol by trained professional health care-workers, and the participants were diagnosed for dementia by two experienced clinical dementia researchers. Complete and reliable information regarding the use of primary health care services was extracted from a national registry and merged with data from the cohort study.

The main weakness of the study is limited information on comorbidity in the study cohort, as the GMHR is only a four-dimensional description of the general health state. In 30% of all visits to the GP the main diagnosis was cardiovascular complaints, and somatic diseases seemed to be a major factor for GP contacts, even if other diseases may have an impact on the patient's decision to see the GP. Further, many additional aspects might explain the frequency of visits to the GP, like self-perceived health or the threshold for appointments (for example availability or transportation) that have not been explored in this study. The study had a dropout rate of 40.2% from baseline to first follow-up. The two main reasons for dropping out are death (18.0%) and non-consent to follow-up (14.6%). Dropouts where slightly older, had a lower functional state and worse general health state. Thus, our study cohort may comprise a selected group of patients, and our findings might not be representative for the general population.

As dementia is a progressive chronic condition that impairs cognitive functioning and the ability of independent living it is reasonable to assume that people with dementia would visit their GP more frequently than people without dementia. However, our findings indicate the opposite, and are in line with a study from the UK, where people with dementia were less likely to visit their GP, and also less likely to have had an outpatient appointment in the last three months, compared with people with depression, disability, and people in good health [22].

Connolly et al. [3] found that 80% of their study population received an annual dementia review at their GP. However, they also found that the reviews were poorly executed, that most lacked a social care review, and that discussions with carers were lacking. The results of the present study are similar to findings in studies on patients with severe mental illnesses that describe a decreased access to primary care in this patient group, and that these patients are undertreated even if they see their GP regularly [23]. A Norwegian study, conducted on the same sample population as this study, found that only 19.5% of 415 participants with dementia had a dementia diagnosis known to themselves, their caregiver, or health care workers at the home care services [11]. However, although there have been studies stating that dementia patients are undertreated for other diseases [7], newer research concludes that there are no differences in treatment [8].

Studies from the USA [24–26] show that intensive follow-up by the municipal health care system of home-dwelling persons with dementia and their relatives reduced the need for home care services and prolonged the time to nursing home admission. This indicates that the course of dementia might be positively influenced by adequate medical follow-up and sufficient support of the patient and his/her relatives. Further research should explore the impact of an increased focus on the cooperation and collaboration between GPs and the municipal care and social services.

## Conclusion

Our study shows that in Norway the home-dwelling elderly with moderate to severe dementia consult their GP less often than persons with mild or no dementia. This could indicate a need for better interaction between the municipal care and social services and general practitioners. Further research should include studies on the quality of the medical follow-up for people with dementia, to see if there is any effect in more thorough and regular medical monitoring of these patients.
